# Expression of the glucose transporters GLUT1, GLUT3, GLUT4 and GLUT12 in human cancer cells

**DOI:** 10.1186/1753-6561-6-S3-P4

**Published:** 2012-06-01

**Authors:** Carly Barron, Evangelia Tsiani, Theodoros Tsakiridis

**Affiliations:** 1 Department of Community Health Science, Brock University, St. Catharines, Ontario, Canada, L2S3A1; 2 Translational Radiation Biology Laboratory, Juravinski Cancer Centre, Hamilton, Ontario, Canada, L84 5C2; 3 Department of Oncology, McMaster University, Hamilton, Ontario, Canada, L8S 4L8

## Background

It is well known that cancer cells display increased glucose uptake and consumption [1]. In a rate-limiting step for glucose metabolism, the glucose transporter (GLUT) proteins facilitate glucose uptake across the plasma membrane. In human cells, 14 different GLUT (1-14) isoforms have been discovered. Of these, GLUT1, 3 and 4 have been extensively studied while the more recently discovered GLUT12 has high relevance to cancer [2]. A comprehensive view of the basal levels of GLUT proteins in cancer is lacking and the exact role of these proteins in cancer cell metabolism is unclear. The present study examined GLUT expression in a number of human cancer cell lines.

## Materials and methods

Real-time PCR was used to quantify mRNA levels of GLUT1, GLUT3, GLUT4 and GLUT12 in lung (A549, NCI-H460, SK-MES-1, H1299), breast (MDA-MB-231, MCF-7), and prostate (PC-3, 22RV1, LNCaP) cancer cells and the normal epithelial cells MRC-5, 184B5 and PNT1A. Protein expression of the glucose transporters was examined by immunoblotting and fluorescence microscopy. The expression of GLUT1 and GLUT3 was immunohistochemically examined in tumors from Balb/c nude mice xenografted with A549, H1299 and PC-3 human cancer cells.

## Results

GLUT1, GLUT3, GLUT4 and GLUT12 mRNA was detected at various levels in all cells examined. Cancer cells were found to have significantly higher expression of GLUT proteins than the corresponding normal epithelial cells at both the mRNA and protein level. Furthermore, GLUT expression in cancer was found to follow an abnormal expression pattern compared to healthy cells. GLUT1 and GLUT3 were detected in vivo in human cell line xenograft tumors.

## Conclusions

These findings describe GLUT expression in a number of cancer cells with different mutations and histologies. The differences in the levels and patterns of glucose transporter expression between normal and cancerous human epithelial cells suggests GLUT proteins may serve as an attractive target for the development of therapeutic agents in lung, breast and prostate cancer.
